# High-resolution calibrated and validated Synthetic Aperture Radar Ocean surface wind data around Australia

**DOI:** 10.1038/s41597-023-02046-w

**Published:** 2023-03-23

**Authors:** Salman Khan, Ian Young, Agustinus Ribal, Mark Hemer

**Affiliations:** 1 Commonwealth Scientific and Industrial Research Organisation, Environment Business Unit, Aspendale, Victoria Australia; 2 Department of Infrastructure Engineering, University of Melbourne, Parkville, Victoria Australia; 3 Department of Mathematics, Faculty of Mathematics and Natural Sciences, Hasanuddin University, Makassar, Indonesia; 4 Commonwealth Scientific and Industrial Research Organisation, Environment Business Unit, Hobart, Tasmania Australia

**Keywords:** Physical oceanography, Physical oceanography

## Abstract

The dataset consists of ocean surface wind speed and direction at 10 m height and 1 km spatial resolution around the wider Australian coastal areas, spanning 4 years (2017 to 2021) of measurements from Sentinel-1 A and B imaging Synthetic Aperture Radar (SAR) platforms. The winds have been derived using a consistent SAR wind retrieval algorithm, processing the full Sentinel-1 archive in this region. The data are appropriately quality controlled, flagged, and archived as NetCDF files representing SAR wind field maps aligned with satellite along-track direction. The data have been calibrated against Metop-A/B Scatterometer buoy-calibrated, wind measurements and examined for potential changes in calibration over the duration of the data. The calibrated data are further validated by comparisons against independent Altimeter (Cryosat-2, Jason-2, Jason-3, and SARAL) wind speeds. Several methods for data access are also listed. The database is potentially useful for offshore industries (oil and gas, fisheries, shipping, offshore wind energy), public recreational activities (fishing, sailing, surfing), and protection and management of coasts and natural habitats.

## Background & Summary

Australia is an island continent surrounded by a vast marine estate whose wide latitudinal variation captures a broad range of extremes in marine surface winds^1^. Offshore industries operating in Australian waters, such as oil and gas, fisheries, shipping, and an emerging wind energy industry, as well as offshore public recreational activities (e.g., fishing, sailing, surfing), require knowledge of ocean wind conditions (amongst other variables) for safe operation and planning. Protection and management of coasts (close to which most of the Australian population lives) and natural habitats also need such information. Ocean surface winds are the driving force for the generation of ocean surface waves, and these atmosphere-ocean interactions also strongly modulate the exchange of heat, momentum, energy, and gases etc. across the marine atmospheric boundary layer. However, offshore *in-situ* measurements (greater than 100 km from shore) of marine winds around Australia, that are openly available, are limited to a single meteorological buoy in the Sub-Antarctic Zone (46.7°S, 142°E), with a non-continuous record spanning several deployments over the last decade^2^. Other records are mostly limited to either coastal locations^3,4^, which are typically confounded by land effects and poorly represent marine winds^5^, or they are collected commercially by offshore industry operators and are not openly available. This scarcity of offshore *in-situ* wind observations has driven high dependency on remotely sensed marine winds in studies validating predictions from Numerical Weather Prediction (NWP) models^5^.

Various satellite platforms such as Scatterometers, Radiometers, and Radar Altimeters (RA) have provided long duration and accurate global ocean surface wind records through several space missions^6–13^. Although these types of satellites provide global, long duration, and high-quality marine wind speed records, their spatial resolution of approx. 10–40 km is more suited for open ocean locations and may not capture the high variability of coastal marine winds at small spatial scales. Closer to the shore they provide limited data as the satellite footprint may also contain responses from land or other man-made structures. The more recent delay-doppler (or Synthetic Aperture Radar - SAR) Altimeters have higher resolution in the along-track direction, which allows them to acquire more valid ocean measurements closer to the coast. However, they typically have a narrow swath of approx. 10 km, and considerable spacing (up to 400 km at the equator) between adjacent tracks^6^.

Imaging SAR satellites are side-looking, high-resolution instruments that can collect data in almost all-weather conditions, day or night. Like a Scatterometer, they are sensitive to ocean surface roughness of cm-scale waves produced by wind stress and can be used to derive ocean surface winds^14^. Several past and present C-band SAR satellites (e.g., ERS-1/2, ENVISAT, Sentinel-1 A/B, Gaofen-3) have collected open ocean data^15–18^, where surface winds can also be computed. Unlike Scatterometers and Radiometers, SAR open ocean observations are discontinuously sampled at low rate and small footprints because of priority given to land coverage for numerous applications^19–22^, and are therefore not ideal for capturing broadscale global ocean wind fields. However, in coastal areas offshore portions of relatively wider-swath (typically 250 – 400 kms) land mode SAR acquisitions, often extending up to several hundred km offshore, can be exploited to produce high resolution (approx. 500 m – 1 km) marine wind maps. Imaging SAR satellites can thus complement the global satellite wind record by capturing spatial variability of coastal wind fields in high resolution.

The ability of SAR satellites to capture high resolution coastal wind fields has driven the development of operational systems to produce coastal SAR wind products at national scales. In North America, the National Oceanic and Atmospheric Administration (NOAA) has developed and operationalised such wind products from Radarsat-2 and Sentinel-1 platforms^23–26^, while in European seas the Technical University of Denmark (DTU) Wind Energy projects have seen the development of a European focused SAR winds database comprising ENVISAT, TerraSAR-X, and Sentinel-1 missions^27–30^. However, such a database has largely been missing in Australia.

This paper outlines a data archive of coastal winds around Australia derived from the Sentinel-1 SAR missions. The archive uses Sentinel-1 level-2 ocean wind product^31^ as input and uses a consistent wind inversion algorithm and geophysical model function to produce quality-controlled SAR winds which are calibrated against Scatterometer (Metop A and B) and validated against independent Altimeter (Cryosat-2, Jason-2, Jason-3, and SARAL) winds. The following sections describe the wind inversion methodology, quality control, calibration, validation, and archiving formats of this database.

## Methods

### SAR data

The archive consists of data from Sentinel-1 A and B SAR missions in Australian coastal areas. In this region, and generally over land, these missions operate in interferometic wide (IW) swath mode, characterised by an approx. 250 km wide swath^17^. The two satellites together cover the Australasian region in a repeated manner roughly every 12 days. Sentinel-1 satellites contain identical SAR instruments operating in C-band, which are sensitive to ocean surface roughness produced by wind stress^32^. Ocean surface winds can be derived in offshore portions of coastal Sentinel-1 images.

Sentinel-1 level-2 IW ocean surface wind product^31^, produced by ESA (European Space Agency), has been used as the input data, and sourced from the Copernicus Australasia Regional Data Hub (www.copernicus.gov.au). The data are free of charge and openly available to all users via Thematic Real-time Environmental Distributed Data Services (THREDDS; https://dapds00.nci.org.au/thredds/catalog/fj7/Copernicus/Sentinel-1/C-SAR/OCN/catalog.html). Although the level 2 product contains winds derived from the Sentinel-1 measurements, the derivations have used various inversion methodologies and/or geophysical model functions over time so the dataset is not homogeneous^33^. In contrast, the new database has used a uniform method to derive ocean surface winds in the Australian region using variables from the level-2 data as input. All the input variables necessary to uniformly reproduce ocean winds are available in the level-2 ESA product. The following input variables have been used in the SAR wind inversion algorithm: incidence angle, satellite heading, ECMWF wind speed and direction, normalised radar cross section (co-polarised and noise corrected), and mask of invalid data - all defined in satellite along-track direction on a curvilinear latitude/longitude grid.

### Wind inversion

SAR ocean wind inversion relies on the sensitivity of radar backscattered power to local surface wind speed and direction similar to a scatterometer^13,14^. The normalised radar cross section (NRCS) has been empirically found to be a function of local 10 m height surface wind speed and direction (*U*_10_) at fixed instrument characteristics (operating frequency and polarisation) and incidence angle^34–37^ - commonly referred to as a Geophysical Model Function (GMF). An NRCS value can be associated with many wind speed and direction pairs^38^. Therefore, to aid the wind inversion process, generally apriori wind information (typically from a model) is used to uniquely determine the wind field.

Several approaches have been used in the literature to invert SAR ocean wind, and a broad overview of these methodologies is given in surveys articles^13,39^. Sentinel-1 winds in the presented database have been derived using the statistical wind retrieval algorithm^40^ (SWRA) combined with CMOD5.N GMF^36^ for VV polarised NRCS, and the wind direction sensitive polarisation ratio model^41^ to be able to also apply the inversion algorithm on the occasional HH polarised Sentinel-1 acquisitions. The overall algorithm has an implementation similar to the recent versions of the Sentinel-1 level-2 ocean wind algorithm^33^. A brief overview of the approach is given below.

The SWRA is based on combining SAR data (NRCS) with co-located model wind fields to retrieve an optimum wind vector, assuming both SAR observations and modelled winds contain errors^40^. Briefly, it aims to find the wind vector components that minimise the following cost function^40^:

1 $${J}_{ij}={\left(\frac{{\sigma }_{{m}_{ij}}^{0}-{\sigma }_{\omega ,\varphi ,{\theta }_{ij}}^{0}}{\Delta \sigma }\right)}^{2}+{\left(\frac{{u}_{{a}_{ij}}-{u}_{\omega ,\varphi }}{\Delta u}\right)}^{2}+{\left(\frac{{v}_{{a}_{ij}}-{v}_{\omega ,\varphi }}{\Delta {\rm{v}}}\right)}^{2}$$

where *ij* are indices to a valid ocean wind cell in the SAR image. The measured SAR NRCS (in dB) and modelled (apriori) wind components are represented by ${\sigma }_{{m}_{ij}}^{0}$ and ${u}_{{a}_{ij}}$, ${v}_{{a}_{ij}}$, respectively. Note, that in the case of HH polarised SAR data, equivalent-VV NRCS is derived using the wind direction sensitive polarisation ratio model^42^ with apriori wind direction as input. A wide range of wind vector field values (wind speed, *ω*, and wind from direction relative to radar look direction, *φ*) decomposed into *u*_*ω,φ*_ and *v*_*ω,φ*_ components are used as trial winds in the cost function. Note, that the radar look direction relative to geographical North, *ψ*, for Sentinel-1 (a right-looking SAR) is given by *ψ* = (*χ* + 90) % 360, where *χ* is the satellite heading with respect to geographical North and % represents the modulo function. Each simulated trial wind is used in CMOD5.N GMF to generate trial NRCS (in dB), ${\sigma }_{\omega ,\varphi ,{\theta }_{ij}}^{0}$, where *θ*_*ij*_ is the incidence angle closest to the measured incidence angle in wind cell *ij*, and is selected from a wide range of values over which the GMF is defined. The term, *lookup table (LUT)*, is commonly used to refer to a range of trial winds, incidence angles, and NRCS over which the cost function is scanned for a minimum value. In the proposed database, the LUT dimensions are as follows:

1. *ω* ranges from 0 to 50 ms^−1^ at 0.1 ms^−1^ intervals,
2. *φ* varies from 0 to 180° with a 0.5° spacing (180° to 360° range is not needed as modelled NRCS is even symmetric around 180°), and
3. *θ* is defined from 28° to 47° with a 0.1° spacing

The standard deviation of errors for SAR observations and apriori winds are modelled by Δ*σ*, Δ*u*, and Δv. Default values of Δ*σ* = 0.1 dB, Δ*u* = 2 ms^−1^, and Δv = 2 ms^−1^ are used^33^. The retrieved wind direction relative to radar look direction, *φ*, is finally converted to meteorological wind direction, *φ*_*M*_ = (*ψ* + *φ*′) % 360, where $$\varphi {\prime} =\left\{\begin{array}{c}360+\varphi ,\varphi  < 0\\ \varphi ,\varphi \ge 0\end{array}\right.$$.

An invalid mask variable inherited from Sentinel-1 level-2 wind data is used to process only valid ocean measurements in a SAR image. The level-2 mask variable already labels *land*, *no_data*, and *sea_ice* as invalid. In the database, any ocean measurement where ${\sigma }_{{m}_{ij}}^{0}\le 0$ is also considered invalid. Because the focus of the proposed database is on coastal ocean data (offshore from land/ocean boundary), SAR wind measurements over inland water bodies, such as lakes, (although possible) are also removed from the database using high-resolution shorelines data from the Global Self-consistent, Hierarchical, High-resolution Geography (GSHHG) Database^42^ (https://www.ngdc.noaa.gov/mgg/shorelines/gshhs.html), and labelled as invalid.

### Quality control

#### Wind inversion quality

The first step in quality control of the inverted SAR wind field is the assessment of wind inversion quality. The ratio of measured NRCS, ${\sigma }_{{m}_{ij}}^{0}$, to simulated NRCS, ${\sigma }_{\omega ,\varphi ,{\theta }_{ij}}^{0}$, (both in linear units) computed using retrieved wind field and CMOD5.N GMF, is a statistic that is representative of the wind inversion quality, ${\sigma }_{{r}_{ij}}^{0}={\sigma }_{{m}_{ij}}^{0}/{\sigma }_{\omega ,\varphi ,{\theta }_{ij}}^{0}$. It is empirically found to follow a unit mean Gaussian distribution, and the outliers can be identified using Median Absolute Deviation^43^ (MAD). The MAD, defined by Huber^44^, can be represented as:

2 $$MAD=b\;median\left\{\left|{\sigma }_{{r}_{ij}}^{0}-{M}_{n}\right|\right\}$$

where ${M}_{n}=median\left\{{\sigma }_{{r}_{ij}}^{0}\right\}$, and *b* has a value of 1.4826, which is the scaling factor for Gaussian distributions^45^.

Wind inversions are labelled as *good*, *medium*, or *bad* quality based on various criteria of deviation of ${\sigma }_{{r}_{ij}}^{0}$ from the median. Outliers are labelled as *bad* inversions, using a threshold of ±3 × *MAD* from median^46^. Inside this threshold, the inversions are considered acceptable and labelled as *good* when the ratio statistic is under the threshold of ±1.5 × *MAD* from the median, and as *medium* otherwise. A summary of formulas for these criteria are listed in Table 1.

**Table 1:** Summary of criteria used to label wind inversions as good, medium, or bad.

**Inversion quality**	**Criteria**
*good*	${M}_{n}-1.5\times MAD < {\sigma }_{{r}_{ij}}^{0} < {M}_{n}+1.5\times MAD$
	or
	$$\left|\frac{{\sigma }_{{r}_{ij}}^{0}-{M}_{n}}{MAD}\right| < 1.5$$
*medium*	${M}_{n}-3\times MAD < {\sigma }_{{r}_{ij}}^{0} < {M}_{n}+3\times MAD$, and ${M}_{n}-1.5\times MAD\ge {\sigma }_{{r}_{ij}}^{0}\ge {M}_{n}+1.5\times MAD$
	or
	$\left|\frac{{\sigma }_{{r}_{ij}}^{0}-{M}_{n}}{MAD}\right| < 3$ and $\left|\frac{{\sigma }_{{r}_{ij}}^{0}-{M}_{n}}{MAD}\right|\ge 1.5$
*bad*	${M}_{n}-3\times MAD\ge {\sigma }_{{r}_{ij}}^{0}\ge {M}_{n}+3\times MAD$
	or
	$$\left|\frac{{\sigma }_{{r}_{ij}}^{0}-{M}_{n}}{MAD}\right|\ge 3$$

#### Wind quality flag

The wind quality flag is derived from the combination of wind inversion quality and percentage of bright targets (*pbright*) detected in the 1 km resolution SAR wind cell. The *pbright* is taken unchanged from the Sentinel-1 level-2 ocean wind product. Three wind quality flag labels are defined following the IMOS standard flag system^47^: *Good_data, Probably_good_data*, and *Bad_data*. When wind inversion quality is acceptable (*good* or *medium*) then the wind quality is considered *Good_data* if *pbright* < = 25%, *Probably_good_data* if *pbright* >25% and < = 50%, otherwise *Bad_data*. When the wind inversion quality is *bad*, then regardless of the value of *pbright*, wind quality is considered *Bad_data*.

#### Product-level geophysical calibration constant: mean, median, and percentile

Three statistics (mean, median, and percentile) of the ratio of measured to simulated NRCS computed over the SAR wind image are also provided. The simulated NRCS is computed in the same manner as done in the *Wind inversion quality* Section, except that ECMWF wind speed and direction are used as inputs to the CMOD5.N GMF instead of the retrieved wind field. These statistics roughly represent the calibration quality of the SAR image product but should be used with caution and only in the most extreme cases of suspected issues, as noted for the mean value in the Sentinel-1 Ocean wind retrieval algorithm description^33^. Because of the sensitivity of the mean to outliers (in this case, e.g., bright targets, frontal systems, wind lulls, and other phenomena in a SAR image), the median value of the ratio is proposed because of its robustness to outliers. In addition, the percentile value of the median statistic relative to the full database of SAR wind products is also provided. The percentile statistic is simpler from a user’s perspective when filtering out SAR wind images with potential calibration issues. Experimental trials suggest that high percentile values (much greater than 99^th^ percentile) of the geophysical calibration constant (median) are associated with bright frontal systems in SAR images, while low values (far lower than 1^st^ percentile) are usually related to SAR images containing wind lulls. In both these extreme cases, the geophysical calibration quality of the SAR wind image product can be questionable because of: (i) SAR signal saturation and dependency of SAR signal on oceanic and atmospheric variables in addition to surface wind (frontal systems), as well as (ii) due to weak (or absence of) SAR signal at the order of (or below) system noise (wind lulls).

### Calibration against scatterometer measurements

The calibration of quality-controlled SAR wind speed data is performed against calibrated Scatterometer wind observations because *in-situ* marine wind observations are limited in the Australian region. The Scatterometer wind database used here has been calibrated against National Data Buoy Centre (NDBC) *in-situ* buoy winds and cross validated^9^. Only Metop-A and B Scatterometers included in the database were found to have observations in close spatial and temporal proximity to Sentinel-1 winds, also termed as *matchups*. The *matchups* satisfied the following criteria:

1. SAR wind measurement was within 50 km and 3 hours of the Scatterometer observation. Usually, in calibration against *in-situ* data a time difference criterion of 30 mins is considered^6,9^, but no matchups were found using this criterion. Several longer matchup intervals were tested (2 hrs, 3 hrs, 4 hrs), and a relatively relaxed interval of 3 hours was chosen as a compromise to increase the number of matchups.
2. Wind speeds which are greater than 60 m/s have been excluded.
3. A minimum of five SAR wind data were required within the spatial collocation (50 km).
4. Large variability in SAR wind speeds were also excluded. Specifically, if $\sigma ({U}_{10})/{\bar{U}}_{10} > 0.2,$ then the matchups were excluded, where *σ*(*U*_10_) and ${\bar{U}}_{10}$ are the standard deviation and mean, respectively, of SAR wind within the spatial collocation.

A linear regression analysis is carried out between SAR and scatterometer wind speed (*U*_10_) matchups. However, because winds from both these types of satellite platforms can contain errors, the linear regression analysis should be modified to take this into account. In such a case, reduced major axis (RMA) regressions can be used^48^. In contrast to a traditional regression, which minimizes the vertical axis offset from the regression line, the RMA regression minimizes the triangular area bounded by the vertical and horizontal offsets between the data point and the regression line and the cord of the regression line. In addition, robust regression^49^ is used because standard least squares regression analysis is highly sensitive to outliers. Robust regression assigns a weight (between 0 and 1) to each data point. Points with a value less than 0.01 are designated as outliers and removed from the analysis before applying the RMA regression analysis.

Calibration performance is evaluated using four statistical parameters, bias *B*, root-mean-square-error (*RMSE*), Pierson’s correlation coefficient (*ρ*), and scatter index (*SI*) defined as follows^6^:

$$B=\frac{1}{N}\mathop{\sum }\limits_{i=1}^{N}\left({M}_{i}-{O}_{i}\right),$$

$$RMSE=\sqrt{\frac{1}{N}\mathop{\sum }\limits_{i=1}^{N}{\left({M}_{i}-{O}_{i}\right)}^{2}},$$

$$SI=\frac{\sqrt{\frac{1}{N}{\sum }_{i=1}^{N}{\left({M}_{i}-{O}_{i}-B\right)}^{2}}}{\frac{1}{N}{\sum }_{i=1}^{N}{O}_{i}},$$

$$\rho =\frac{{\rm{cov}}(M,O)}{{\sigma }_{M}{\sigma }_{O}},$$

where *M* and *O* represent reference (Scatterometer) and SAR measurements, respectively, *N* is the number of matchup points, *σ* is the standard deviation, and cov is the sample covariance.

**Fig. 1:**
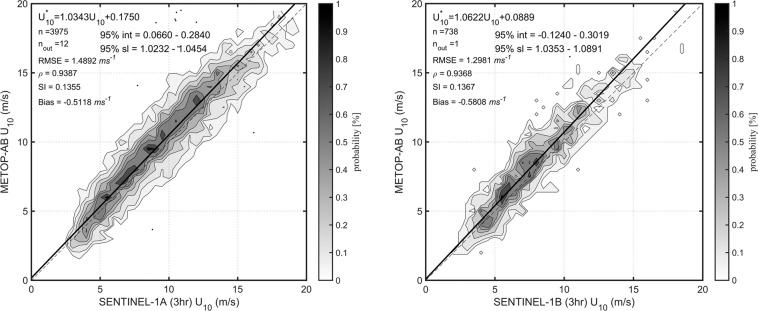
Calibration of Sentinel-1 wind speed against Metop data. Shown are the 1:1 agreement (dashed diagonal line) and the RMA regression (thick solid line). Contours show the density of matchup data points, which has been normalized such that the maximum value is 1.0. Contours are drawn at 0.9, 0.7, 0.5, 0.4, 0.3, 0.2, 0.1, and 0.05. Dots represent outliers excluded from the RMA regression.

The buoy-calibrated wind speeds of the two Metop Scatterometers are similar and have been verified through cross validation^9^. Therefore, for each Sentinel-1 platform, matchup data across both Metop Scatterometers are pooled together to increase the number of matchups for calibration. The calibration results show that the SAR *U*_10_ values match well with Scatterometer data with only slight deviations from the 1:1 agreement line (Fig. 1). Both Sentinel-1 platforms yield overall slightly lower wind speeds than matching scatterometer data. Similar results (not shown) were also obtained using different matchup intervals or without pooling together the data from Metop platforms. These results agree with the preliminary evaluation of Sentinel-1 winds against Metop data^32^.

**Fig. 2:**
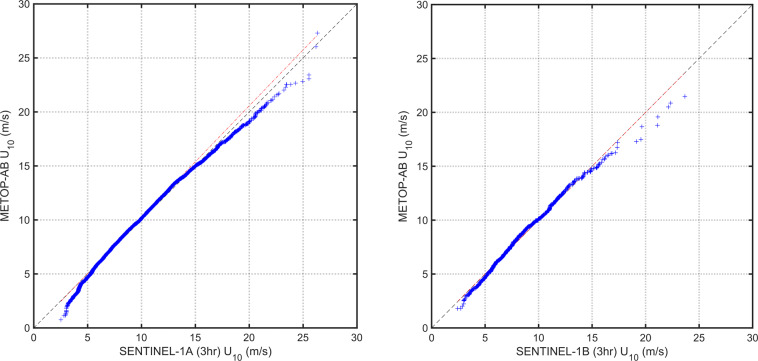
Q–Q plots between the SAR and Metop-A/B wind data matchups after the calibrations were applied.

The performance at high and low wind speeds can be understood by examining Q-Q plots (Fig. 2) after applying the linear calibration adjustment to SAR wind speed. At high (>15 ms^−1^) and low winds (<4 ms^−1^), Sentinel-1 wind speeds are overestimated compared to Scatterometer data. In some recent works^9,50^ a separate empirical correction has been applied for similar behaviour in high winds shown by Radiometer and Scatterometer measurements. In the proposed database, such a correction has not been applied, but could be considered in future developments. The final SAR linear calibration relations are summarized in Table 2.

**Table 2:** Calibration relationships for SAR wind speed, obtained from the RMA regression.

SAR	Period	Calibration relation	95% limit slope	95% limit offset	n	Percent outliers
S1A	24/10/2017 – 31/08/2021	${U}_{10}^{\ast }$ = 1.0343*U*_10_ + 0.175	1.0232–1.0454	0.066–0.248	3,975	0.3
S1B	as above	${U}_{10}^{\ast }$ = 1.0622*U*_10_ + 0.0889	1.0353–1.0891	−0.124–0.3019	738	0.135

${U}_{10}^{\ast }$ is the calibrated value and *U*_10_ is the uncalibrated data. Also shown are the confidence limits on the regression, number of points n, and the percentage of outliers from the robust regression.

**Fig. 3:**
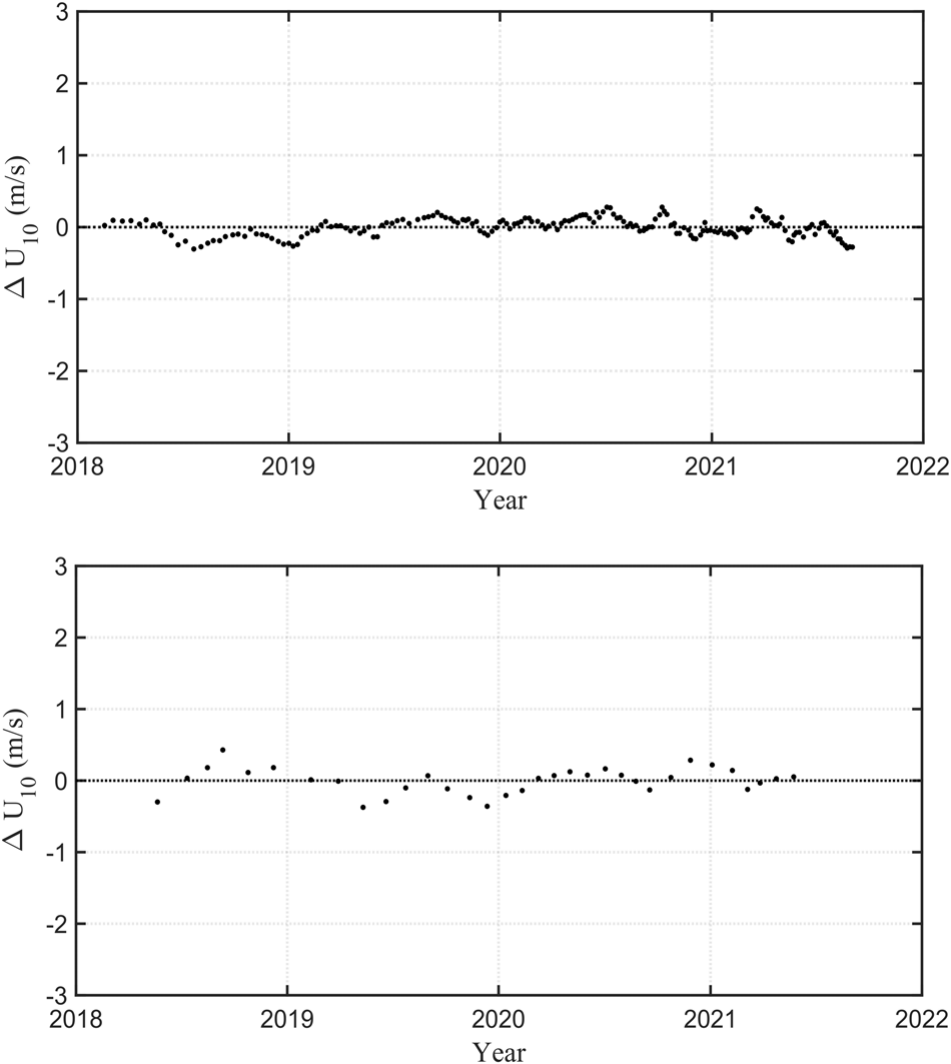
Difference between Sentinel-1 and Metop values of *U*_10_ as a function of time after applying calibration over the full period of the mission. Sentinel-1 A top and Sentinel-1 B bottom panel.

The above calibration procedure can be regarded as an *average* calibration over the full SAR data duration. It doesn’t reveal any changes in calibration over time, e.g., due to satellite drift or discontinuities in calibration. Such changes can be evaluated by examining the differences between Metop and SAR (calibrated) wind speeds as a function of time (Fig. 3). The analysis reveals that there is no significant change in calibration over time for both the Sentinel-1 platforms.

**Fig. 4:**
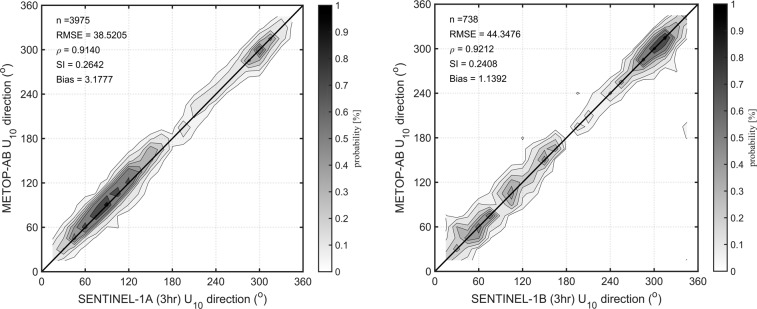
Comparison between Sentinel-1 and Metop wind direction. The 1:1 agreement line is shown (thick solid line). Contours show the density of matchup data points, which has been normalized such that the maximum value is 1.0. Contours are drawn at 0.9, 0.7, 0.5, 0.4, 0.3, 0.2, 0.1, and 0.05.

Comparisons of wind direction between Sentinel-1 and Metop platforms was also carried out. The same collocation criteria as for wind speed calibration were used, i.e., SAR measurement within 50 km and 3 hours of the Metop observation. For both Sentinel-1 platforms excellent agreement with Scatterometer wind direction was observed (Fig. 4), and therefore no modifications or calibrations were applied to Sentinel-1 wind directions.

## Data Records

A static “snapshot” of the data as described in this paper has been archived at the Australian Ocean Data Network (AODN) repository^51^ – which is the main repository of ocean observations in Australia. This is a full copy of all data at the date of submission of this publication.

**Fig. 5:**
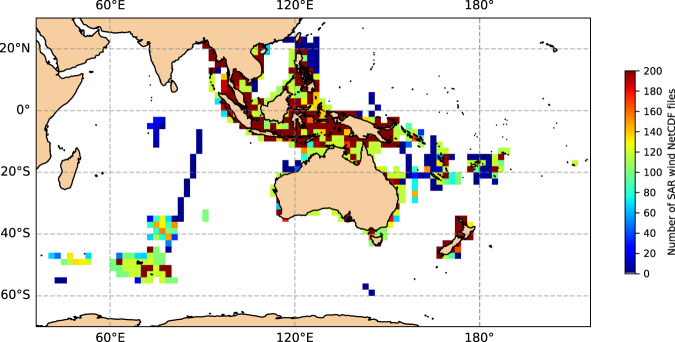
Geographical coverage and spatial distribution of combined Sentinel-1 A and B coastal wind database. Total number of wind NetCDF files falling into 2° × 2^o^ lat/lon bins is shown. Only mean lat/lon values are used in the binning process.

A total of 16 variables are stored in the database records (Table 3). Each NetCDF file represents a portion of the along-track satellite swath recorded on an irregular latitude/longitude grid, similar to the source ESA level-2 product, with dimensions of TIME (in along-track direction) and RANGE (in cross-track direction). This arrangement ensures that each NetCDF file is not too large and still captures small-scale (~ 1 km) spatial variability of wind field. Valid wind cells in a NetCDF file can be selected using the mask variable. Multiple NetCDF files of the same swath can also be analysed together to study larger geographical areas. A global attribute, *percent_coverage*, is provided to filter out NetCDF files with little or no wind data. The database commences from Oct 2017 and is up to date till Aug 2021 at the time of writing this article and covers an Australasian coastal region of interest (Fig. 5). The sources of the various variables are described below.

- LATITUDE, LONGITUDE, INC_ANGLE, PBRIGHT, WSPD_ECMWF, and WDIR_ECMWF are unchanged from Sentinel-1 level-2 ocean wind NetCDF, except that LONGITUDE is translated to 0°–360° range.
- TIME is extracted from Sentinel-1 level-2 ocean wind. SAFE file name.
- NRCS_VV and MASK have been explained previously in the *Wind inversion* Section.
- AZIMUTH is computed as 90° clockwise from satellite heading, which is extracted from Sentinel-1 level-2 ocean wind NetCDF
- INV_QUALITY has been explained previously in *Wind inversion quality* Section.
- WSPD and WDIR are the inverted SAR wind speed and direction
- WSPD_CAL is the calibrated SAR wind speed
- WSPD_quality_control and WSPD_CAL_quality_control are IMOS convention wind quality flags for raw and calibrated SAR wind speed

**Table 3:** List of all variables included in the database.

No.	NetCDF variable name	Description
1	TIME	Time
2	LATITUDE	Latitude
3	LONGITUDE	Longitude
4	MASK	Mask of invalid data (0-valid, 1-invalid)
5	NRCS_VV	Calibrated and noise-corrected, equivalent-VV normalised radar cross section
6	AZIMUTH	Radar look direction relative to North
7	INC_ANGLE	Incidence angle
8	PBRIGHT	Percentage of bright targets detected in wind cell
9	INV_QUALITY	Quality of wind inversion: good (0), medium (1), bad (2)
10	WSPD_ECMWF	ECMWF wind speed
11	WDIR_ECMWF	ECMWF wind direction
12	WSPD	SAR wind speed at 10 m height assuming neutral marine boundary layer
13	WDIR	SAR wind from direction at 10 m height, measured positive clockwise from due North
14	WSPD_quality_control	Wind quality flag
15	WSPD_CAL	Calibrated SAR wind speed at 10 m height
16	WSPD_CAL_quality_control	Calibrated wind quality flag

All data files are provided in NetCDF format following IMOS data standards^47,52^ upon which the project is based. The IMOS standard flag system is used for all data flags – where flag values of 1, 2, and 4 represent *Good_data*, *Probably_good_data*, and *Bad_data*, respectively. The filenames follow the format:

**IMOS_SRS-Surface-Waves_M_YYYYMMDD_Coastal-Wind-SAR_FV01_DM00-AbsoluteOrbitNumber-DataTakeId-ProductId.nc**where

- IMOS: name of the project.
- SRS-Surface-Waves: representing the present facility.
- M: signifies meteorological related parameters.
- YYYYMMDD: Start date of the observation.
- Coastal-Wind-SAR: Coastal wind from Sentinel-1A or B (variable), i.e., Coastal-Wind-Sentinel-1A or Coastal-Wind-Sentinel-1B.
- FV01: representing file version.
- DM00-AbsoluteOrbitNumber-DataTakeId-ProductId: Unique product reference - a combination of version of delayed mode product (DM00), 6-digit absolute orbit number, 6-digit data take id (hexadecimal), 4-digit product id (hexadecimal).

There are approximately more than 90,000 NetCDF files in the full combined Sentinel-1 A and B database, which have been stored in the following folder hierarchy:

/Satellite_Name/YYYY/MM/DD

e.g., /Sentinel-1A/2021/08/01/IMOS_SRS-Surface-Waves_M_20210801_Coastal-Wind-Sentinel-1A_FV01_DM00-039029-049AF1-02FA.nc

A dynamic archive is also maintained at the AODN Portal (https://portal.aodn.org.au/) as it is intended that the database will be extended at approximately 6-month intervals. The user can access the data in the following ways:

(i) Graphical user interface at the AODN portal (https://portal.aodn.org.au/search?uuid=b02b929f-2caf-45d4-ac60-d4632b7ca0ca)
(ii) Amazon S3 server (http://data.aodn.org.au/?prefix=IMOS/SRS/Surface-Waves/SAR_Wind/)
(iii) AODN THREDDS server (https://thredds.aodn.org.au/thredds/catalog/IMOS/SRS/Surface-Waves/SAR_Wind/catalog.html)

## Technical Validation

The validation of calibrated SAR wind speed data is conducted against an independent Altimeter derived wind database^6,7^. The altimeter wind speeds have been calibrated against NDBC buoy winds, cross validated amongst altimeters, and used in several global studies^12,50,53^.

The criteria for obtaining SAR-Altimeter matchups are the same as for SAR matchups with Metop Scatterometers, i.e., Altimeter wind observations within 50 km and 3 hours of SAR measurements are considered as matchups. Using these criteria Cryosat-2, Jason-2, Jason-3, and SARAL were the only altimeters that had matchups with Sentinel-1 wind data: 476 matchups for Sentinel-1 A and 126 for Sentinel-1 B.

The comparisons are performed using robust RMA regression analysis as done previously during the calibration process. Again, the reasons are that conventional linear regression (as opposed to robust regression) is sensitive to outliers and doesn’t account for potential errors in both datasets. The outliers are removed prior to performing RMA regression.

**Fig. 6:**
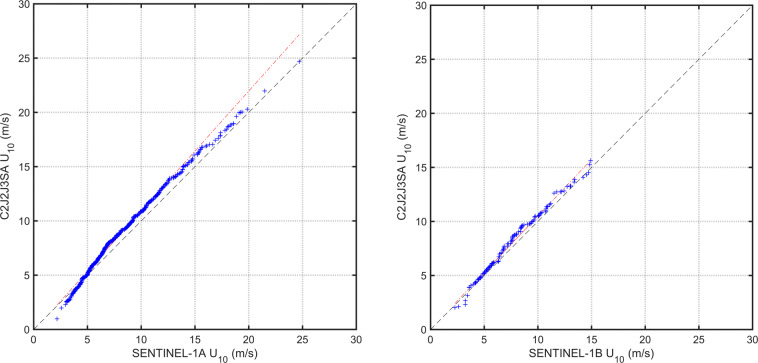
Q–Q plots between the calibrated Sentinel-1 and Altimeter data for wind speed. The abbreviations of C2, J2, J3, SA have been used for Cryosat-2, Jason-2, Jason-3, and SARAL, respectively.

Q-Q plots of the comparison are shown in Fig. 6. It is clear from the results of regression analysis that the calibrated SAR wind speeds match well with Altimeter wind speeds with only slight deviations. Considering that these two datasets have been obtained from two completely different instrument types (SAR vs Altimeter) with independent data processing, and that a relatively small sample size of matchups is obtained, the comparisons are reasonably convincing and provide adequate validation of the accuracy of SAR wind speeds in the proposed database.

## Usage Notes

**Fig. 7:**
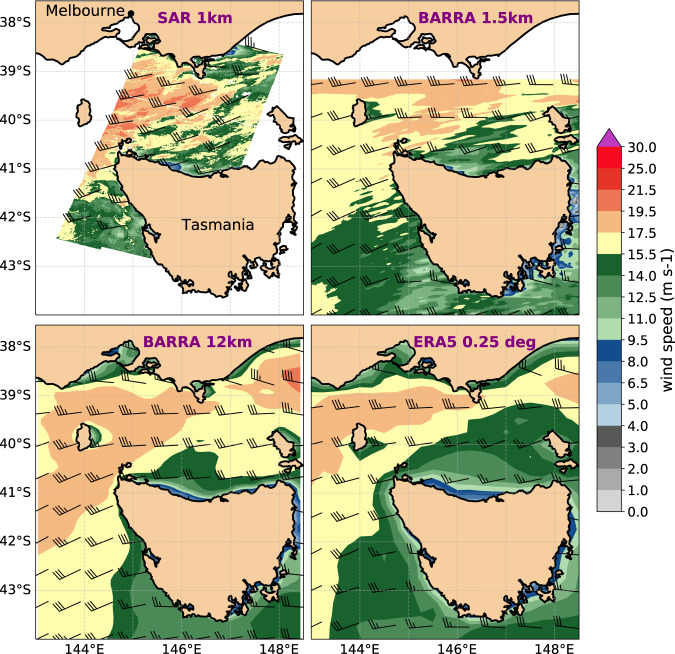
Sentinel-1 SAR descending pass capturing strong westerly marine surface winds in the Bass Strait that contributed to damaging conditions in parts of Southern Victoria on 14 April 2018 and its visual comparison with reanalysis winds at various spatial resolutions. (top-left) SAR wind map at approximately 19:25:00 utc and 1 km resolution, (top-right) BARRA high resolution (1.5 km) wind field, (bottom-left) BARRA lower resolution (12 km) wind field both at 19:30:00 utc, and (bottom-right) ERA5 surface wind field (0.25 deg horizontal resolution) at 19:00:00 utc.

A sample use of the data, alongside software code (see Code Availability), is provided showing the SAR winds product capturing a strong winds event in Southeast Australia. Wind field from several NetCDF files of a descending Sentinel-1 pass covering a portion of the Bass Strait are plotted together, and the product is visually compared with wind maps from Bureau of Meteorology Atmospheric high-resolution Regional Reanalysis for Australia^54,55^ (BARRA) at two resolutions 1.5 km and 12 km, and ECMWF Reanalysis v5^56,57^ (ERA5) at 0.25 deg. horizontal resolution (Fig. 7). Similarities in the spatial distribution of observed and reanalysis wind fields can be spotted, especially when compared with the high-resolution BARRA product.

The data can be analysed using a variety of software for manipulating and displaying NetCDF files (see https://www.unidata.ucar.edu/software/netcdf/software.html). Python notebooks with numpy, xarray, matplotlib, and cartopy packages are recommended for analysing the data.

## Data Availability

A Python Jupyter notebook for getting started with reading the data and comparing them with other reanalyses datasets at matching times (as outlined in the Usage Notes Section) is available at the AODN GitHub repository (https://github.com/aodn/imos-user-code-library/blob/master/Python/notebooks/SAR_winds/SAR_winds_getting_started_jupyter_notebook/ausar_winds_getting_started_notebook.ipynb).
